# Jinghua Weikang capsule for *helicobacter pylori* eradication: A systematic review and meta-analysis with trial sequential analysis

**DOI:** 10.3389/fphar.2022.959184

**Published:** 2022-09-26

**Authors:** Qian Zhao, Wen-jia Wang, Shui-ping Zhou, Jing Su, He Sun, Jing-bo Zhai, Yun-hui Hu

**Affiliations:** ^1^ Cloudphar Pharmaceuticals Co, Ltd., Shenzhen, China; ^2^ The State Key Laboratory of Core Technology in Innovative Chinese Medicine, Tasly Academy, Tasly Holding Group Co, Ltd., Tianjin, China; ^3^ Tasly Pharmaceutical Group Co, Ltd., Tianjin, China; ^4^ School of Public Health, Tianjin University of Traditional Chinese Medicine, Tianjin, China

**Keywords:** Jinghua Weikang capsule, *Helicobacter pylori* eradication, triple therapy, quadruple therapy, systematic review

## Abstract

**Background:** *Helicobacter pylori* (*H. pylori*) infection is one of the most common chronic bacterial infections worldwide. The resistance of *H. pylori* to antibiotics may increase the risk of treatment failure. Complementary and alternative regimens are still needed. This study aimed to critically assess the efficacy and safety of Jinghua Weikang capsule (JWC) for *H. pylori* eradication.

**Materials and methods:** PubMed, Embase, Web of Science, Cochrane library, China National Knowledge Infrastructure, Wanfang Digital Periodicals, and Chinese Science and Technology Periodicals database were searched from inception to April 2022. Randomized controlled trials (RCTs) comparing a combination of JWC and conventional treatments with conventional treatments alone or combined with a placebo for *H. pylori* eradication were considered for inclusion. The primary outcome was *H. pylori* eradication rate. The meta-analysis and trial sequential analysis (TSA) were conducted where possible.

**Results:** A total of 34 studies were included in the statistical analysis. A pooled result showed that JWC with the duration of 2 weeks combined with the triple/quadruple therapy could significantly increase the *H. pylori* eradication rate compared with the triple/quadruple therapy alone (RR: 1.13, 95% CI: 1.05 to 1.21, *p* = 0.0008). However, the evidence of benefit was not confirmed by TSA. Another pooled result showed that JWC with the duration of 4 weeks combined with the triple/quadruple therapy could significantly increase the *H. pylori* eradication rate compared with the triple/quadruple therapy alone (RR: 1.21, 95% CI: 1.15 to 1.27, *p* < 0.00001). The evidence of benefit was confirmed by TSA. There were no statistically significant differences in the incidence of adverse reactions between the two groups.

**Conclusion:** The present study suggests that JWC with the duration of 4 weeks can significantly improve the *H. pylori* eradication rate and should be considered as a complementary treatment to conventional regimens for *H. pylori* eradication. However, more high-quality RCTs are still needed to confirm these findings.

## 1 Introduction

*Helicobacter pylori* (*H. pylori*) belongs to gram-negative bacteria (IARC Working Group on the Evaluation of Carcinogenic Risks to Humans, 2012). *H. pylori* infection is one of the most common chronic bacterial infections worldwide (Randel, 2018). More than 50% of the population is infected by *H. pylori* in the world. However, the prevalence of *H. pylori* infection varies across regions and countries (Hooi et al., 2017). *H. pylori* infection may be associated with multiple factors, such as socioeconomic status and health care resources (Hooi et al., 2017). It may contribute to some gastrointestinal diseases, such as gastritis and peptic ulcer (Malfertheiner et al., 2007; Malfertheiner et al., 2017). *H. pylori* has been classified as carcinogenic to humans by the International Agency for Research on Cancer (IARC Working Group on the Evaluation of Carcinogenic Risks to Humans, 2012). It can increase the risk of gastric cancer (Torre et al., 2015; Machlowska et al., 2020). However, a systematic review has shown that the incidence of gastric cancer can be significantly reduced by eradicating *H. pylori* (Lee et al., 2016).

Many drugs can be used for *H. pylori* eradication, such as proton pump inhibitors (PPIs), clarithromycin, amoxicillin, and metronidazole. Multiple combinations of these drugs, such as clarithromycin triple therapy and bismuth quadruple therapy, have been recommended for eradicating *H. pylori* according to practice guidelines from the American College of Gastroenterology (Chey et al., 2017). A recent systematic review showed that the pooled prevalence rate of *H. pylori* resistance to clarithromycin, metronidazole, or levofloxacin was more than 15% (Savoldi et al., 2018). It is noteworthy that the resistance of *H. pylori* to antibiotics may increase the risk of treatment failure (Chey et al., 2017; Shiotani et al., 2017). However, the development of new antibiotics has not met the needs for gram-negative organism eradication at present (Laxminarayan et al., 2020). Therefore, complementary and alternative regimens are still needed (Savoldi et al., 2018).

Traditional Chinese medicine (TCM) has been attracting attention for *H. pylori* eradication (Li et al., 2021a; Li et al., 2021b; Zhong et al., 2022). Jinghua Weikang capsule (JWC) as a specific TCM is composed of *Dysphania ambrosioides* (L.) Mosyakin & Clemants and *Adina pilulifera* (Lam.) Franch. ex Drake described in Table 1 (Chen, 2005; Zhu et al., 2012; Shi et al., 2018). Some randomized controlled trials (RCTs) have investigated the efficacy of JWC for *H. pylori* eradication (Zhou, 2008; Zhang, 2012a; Bai, 2012; Zhang, 2012b; Zhang Y., 2013a). The results showed that JWC might be beneficial for *H. pylori* eradication. However, these trials have a relatively small sample size and have been not comprehensively searched and combined to increase the power and improve the precision of the estimated intervention effects due to the lack of a high-quality systematic review on this topic. Therefore, this systematic review was conducted to critically assess the efficacy and safety of JWC for *H. pylori* eradication.

**TABLE 1:** Main components of the Jinghua Weikang capsule.

Formulation	Source	Species, family, genus	Quality control reported (Y/N)	Chemical analysis reported (Y/N)
Jinghua Weikang capsule	Tasly pharmaceutical group Co., Ltd, Tianjin, China	1. *Dysphania ambrosioides* (L.) Mosyakin & Clemants	Y—Prepared according to the state food and drug administration, national drug standards [WS3-404 (Z-058)-2001(Z)-2007]	Y—Chen, 2005; Zhu et al., 2012
		Family: *Amaranthaceae* Juss		
		Genus: *Dysphania* R.Br		
		*2. Adina pilulifera* (Lam.) Franch. ex Drake		
		Family: *Rubiaceae* Juss		
		Genus: *Adina* Salisb		

## 2 Materials and methods

This study was registered on PROSPERO (No. CRD42022315488) available from: https://www.crd.york.ac.uk/prospero/display_record.php?ID=CRD42022315488. It was conducted following the Preferred Reporting Items for Systematic reviews and Meta-Analyses (PRISMA) statement (Page et al., 2021).

### 2.1 Inclusion and exclusion criteria

#### 2.1.1 Type of included studies

RCTs were considered for inclusion, regardless of publication date and language. Abstracts, letters, and comments were deleted.

#### 2.1.2 Patients

*H. pylori*-infected patients were included, regardless of age, gender, race, or nationality. Gastrointestinal diseases, such as gastritis and peptic ulcer, were unlimited because they may be associated with *H. pylori* infection. *H. pylori* infection should be tested by internationally recognized methods, such as endoscopic biopsy and a urea breath test (Randel, 2018).

#### 2.1.3 Interventions

A combination of JWC and conventional treatments was used in the experimental group. Comparator interventions included conventional treatments alone or combined with a placebo. Dosage, frequency, and duration were unlimited. Conventional treatments refer to regimens recommended for *H. pylori* eradication by clinical guidelines, such as clarithromycin triple therapy and bismuth quadruple therapy (Randel, 2018).

#### 2.1.4 Outcomes

The primary outcome was *H. pylori* eradication rate. The secondary outcomes included *H. pylori* recurrence rate, cure rate, response rate, and incidence of adverse reactions (such as nausea, diarrhea, dizziness, and constipation). The cure rate was expressed as a percentage of the number of well-healed patients with gastritis or peptic ulcer divided by the total number of patients in a certain group. The cure was defined as the disappearance of clinical symptoms associated with gastritis or peptic ulcer. The response rate was expressed as a percentage of the number of patients meeting the “response” standard divided by the total number of patients in a certain group. The response was defined as more than 50% reduction of peptic ulcer area.

### 2.2 Search strategy

PubMed, Embase, Web of Science, Cochrane library, China National Knowledge Infrastructure, Wanfang Digital Periodicals, and Chinese Science and Technology Periodicals database were searched from inception to April 2022 independently by two reviewers (J. Zhai and Q. Zhao). The search terms included (“*Helicobacter pylori*” OR “*H. pylori*” OR “*Helicobacter* infection” OR Hp) AND (Jinghuaweikang OR “Jinghua weikang” OR “Jing Hua Wei Kang”). The detailed search strategies are available in Supplementary Material. Some clinical trial registry platforms (e.g., ClinicalTrials.gov, World Health Organization International Clinical Trials Registry platform, and Chinese Clinical Trial Registry) and references of eligible studies were also searched. Publication date and language were unlimited.

### 2.3 Study selection

Potentially eligible studies were collected from the comprehensive literature search and imported into EndNote software to remove duplicate studies. Then, two reviewers (J. Zhai and Q. Zhao) independently deleted ineligible studies by checking titles and abstracts according to the inclusion and exclusion criteria. Full texts of the remaining studies were read to identify included studies. The process of screening eligible studies was presented by the PRISMA flow diagram. Disagreements were handled in consultation with a third reviewer (Y. Hu).

### 2.4 Data extraction

The important data were extracted and imported into Excel software by two authors (J. Zhai and Q. Zhao) independently. They included characteristics of included studies (first author, publication year, country, sample size, design), patients (age, gender, race, nationality), interventions (type, dosage, frequency, duration), and outcomes (primary and secondary outcomes). The information on the risk of bias assessment (randomization, allocation, blinding, loss to follow-up) was also extracted synchronously.

### 2.5 Assessment of risk of bias and quality of evidence

The risk of bias was assessed using the Cochrane “risk of bias” tool (Higgins et al., 2022). It can be used to investigate some important biases in clinical trials, such as selection bias, performance bias, detection bias, attrition bias, and reporting bias. The risk of bias was judged to be low, high, or unclear against the judgment of two reviewers (J. Zhai and Q. Zhao) independently. The quality of evidence was assessed using the Grading of Recommendations Assessment, Development, and Evaluation (GRADE) system. Disagreements were resolved by consensus or consultation with a third author (Y. Hu).

### 2.6 Statistical analysis

A risk ratio (RR) with 95% confidence intervals (CIs) was used to estimate the effect of the dichotomous variables. The meta-analysis with the random-effect model was conducted by Review Manager 5.4 software. *p* < 0.05 indicated a statistically significant difference between the two groups. Subgroup analyses were conducted based on control interventions (triple and quadruple therapy) and types of diseases (gastritis and peptic ulcer), if possible. Traditional meta-analysis may lead to the falsely positive or falsely negative conclusions because of sparse data and repeated testing of significance (Wetterslev et al., 2017). Trial sequential analysis (TSA) can be used to evaluate if the evidence from the meta-analysis is sufficiently reliable based on some important parameters (Wetterslev et al., 2008; Thorlund et al., 2009). For the primary outcome, TSA was conducted with a relative risk reduction (RRR) of 10%, type I error of 5% (two-sided), and type II error of 20% (a power of 80%) according to previous studies (Zhang et al., 2015; Zhou et al., 2019). Firm evidence is reached when the cumulative Z-curve crosses the trial sequential monitoring boundary or the futility boundary. Otherwise, it is insufficient to draw any firm conclusion. The publication bias was assessed for the primary outcome by Stata 16 software if the meta-analysis included more than 10 studies.

## 3 Results

### 3.1 Literature search

**FIGURE 1:**
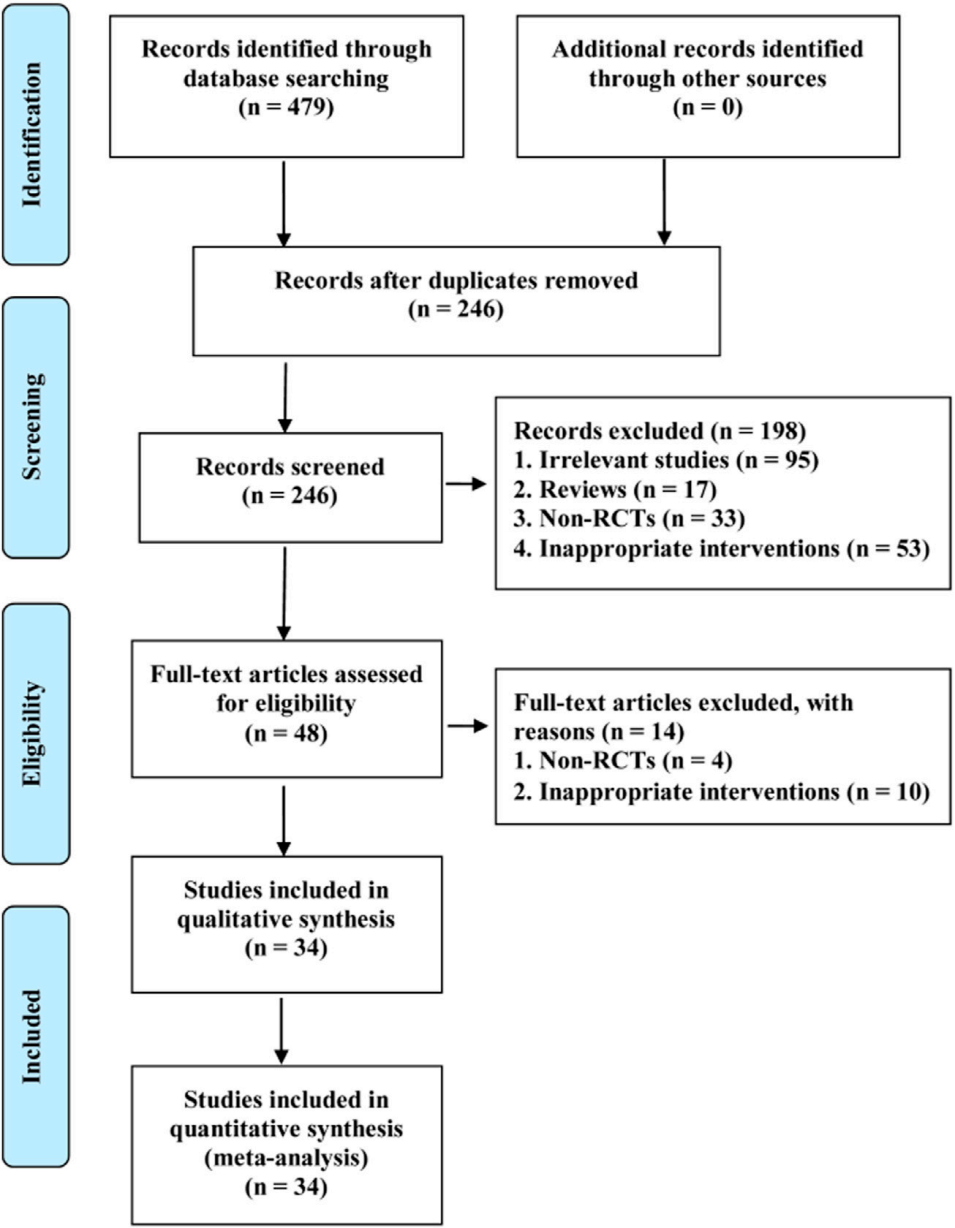
PRISMA flow diagram of study retrieval and selection.

A total of 479 potentially eligible studies were identified from the initial search. Two hundred and thirty-three duplicate studies were removed using EndNote software. One hundred and ninety-eight irrelevant studies were deleted by checking the titles and abstracts. After reading full texts of the remaining records, 34 studies were included in the statistical analysis (Zhou, 2008; Zhang, 2012a; Bai, 2012; Zhang, 2012b; Zhang, 2013a; Zhang., 2013b; Shi and Sun, 2014; Lin, 2015; Wang and Han, 2015; Zhao, 2015; Chai, 2016; Deng, 2016; Lin, 2016; Xu et al., 2016; Su and Wu, 2017; Tao et al., 2017; Wang, 2018a; Wang, 2018b; Chen, 2018; Zhang et al., 2018; Zhi and Jiao, 2018; Dai and Cheng, 2019; Liu, 2019; Niu, 2019; Xu and Yu, 2019; Zhu, 2019; Cheng et al., 2020; Hang, 2020; Yao et al., 2020; Zheng, 2020; Cen, 2021; Wang et al., 2021; Xiong et al., 2021; Zhu, 2021). The process of screening eligible studies is presented in Figure 1.

### 3.2 Characteristics of included studies

The characteristics of the included studies are summarized in Table 2. The included studies involving 3920 patients were published between 2008 and 2021. The sample size ranged from 30 to 138 in the experimental group and 30 to 142 in the control group. JWC plus the triple therapy was compared with the triple therapy alone in 24 trials (Zhou, 2008; Zhang, 2012a; Bai, 2012; Zhang, 2012b; Zhang, 2013a; Zhang, 2013b; Shi and Sun, 2014; Lin, 2015; Wang and Han, 2015; Zhao, 2015; Chai, 2016; Deng, 2016; Lin, 2016; Xu et al., 2016; Su and Wu, 2017; Tao et al., 2017; Wang, 2018a; Wang, 2018b; Zhi and Jiao, 2018; Liu, 2019; Niu, 2019; Cheng et al., 2020; Cen, 2021; Xiong et al., 2021). One trial compared JWC plus the triple therapy with the triple therapy plus placebo (Dai and Cheng, 2019). JWC plus the quadruple therapy was compared with the quadruple therapy alone in nine studies (Chen, 2018; Zhang et al., 2018; Xu and Yu, 2019; Zhu, 2019; Hang, 2020; Yao et al., 2020; Zheng, 2020; Wang et al., 2021; Zhu, 2021). The duration of JWC treatment ranged from 10 days to 8 weeks. Patients with gastritis were enrolled in 14 trials (Bai, 2012; Lin, 2015; Deng, 2016; Xu et al., 2016; Tao et al., 2017; Wang, 2018b; Chen, 2018; Zhang et al., 2018; Dai and Cheng, 2019; Liu, 2019; Hang, 2020; Zheng, 2020; Wang et al., 2021; Zhu, 2021). Patients with peptic ulcer were included in 19 trials (Zhou, 2008; Zhang, 2012a; Zhang, 2012b; Zhang, 2013a; Zhang, 2013b; Shi and Sun, 2014; Wang and Han, 2015; Zhao, 2015; Chai, 2016; Su and Wu, 2017; Wang, 2018a; Chen, 2018; Zhi and Jiao, 2018; Niu, 2019; Xu and Yu, 2019; Zhu, 2019; Cheng et al., 2020; Cen, 2021; Xiong et al., 2021). Two trials recruited patients with gastritis or peptic ulcer (Lin, 2016; Yao et al., 2020). Thirteen studies reported adverse reactions (Bai, 2012; Shi and Sun, 2014; Wang and Han, 2015; Chai, 2016; Wang, 2018b; Liu, 2019; Xu and Yu, 2019; Cheng et al., 2020; Yao et al., 2020; Zheng, 2020; Wang et al., 2021; Xiong et al., 2021; Zhu, 2021).

**TABLE 2:** Characteristics of included studies.

Author and publication year	Gastrointestinal disease	Age (E)	Age (C)	Male (E/C)	Female (E/C)	Sample size (E)	Sample size (C)	Interventions (E)	Interventions (C)	Dosage of JWC	Duration of JWC	Duration of TT/QT	Outcomes
Zhou (2008)	Peptic ulcer	18 - 63	16 - 68	45/40	15/20	60	60	JWC + TT	TT	480 mg/d	4 weeks	Clarithromycin + Amoxicillin 1 week, Omeprazole 4 weeks	HER, CR, RESR
Bai (2012)	Gastritis	53.5	51.7	44/37	33/34	75	70	JWC + TT	TT	480 mg/d	4 weeks	Omeprazole + Amoxicillin + Clarithromycin 4 weeks	HER, AR
Zhang (2012a)	Peptic ulcer	-	-	27/28	23/22	50	50	JWC + TT	TT	480 mg/d	4 weeks	Clarithromycin + Amoxicillin + Omeprazole 4 weeks	HER
Zhang (2012b)	Peptic ulcer	-	-	72	48	60	60	JWC + TT	TT	480 mg/d	4 weeks	Amoxicillin + Tinidazole 10 days, Omeprazole 4 weeks	HER, CR, RESR
Zhang (2013a)	Peptic ulcer	43.12	41.86	25/26	21/20	46	46	JWC + TT	TT	480 mg/d	4 weeks	Amoxicillin + Metronidazole 1 week, Omeprazole 4 weeks	HER, CR, RESR
Zhang (2013b)	Peptic ulcer	35	34	24/26	18/16	42	42	JWC + TT	TT	480 mg/d	4 weeks	Clarithromycin + Amoxicillin 1 week, Omeprazole 4 weeks	HER, CR, RESR
Shi and Sun (2014)	Peptic ulcer	34.8 ± 12.16	36.2 ± 10.53	22/21	20/19	42	40	JWC + TT	TT	480 mg/d	4 weeks	Clarithromycin + Amoxicillin 2 weeks, Lansoprazole 4 weeks	HER, CR, RESR, AR
Lin (2015)	Gastritis	40.53 ± 7.38	39.87 ± 7.37	28/33	58/62	100	100	JWC + TT	TT	480 mg/d	14 days	Omeprazole + Clarithromycin + Amoxicillin 2 weeks	HER, CR
Wang and Han (2015)	Peptic ulcer	15 - 72	43.5	228	52	138	142	JWC + TT	TT	480 mg/d	6 weeks	Clarithromycin + Amoxicillin 1 week, Omeprazole 6 weeks	HER, AR
Zhao (2015)	Peptic ulcer	46.2 ± 3.3	45.6 ± 2.3	37/36	12/13	49	49	JWC + TT	TT	480 mg/d	2 weeks	Amoxicillin + Levofloxacin 2 weeks, Lansoprazole 5 weeks	HER, CR, RESR
Chai (2016)	Peptic ulcer	45.98 ± 5.76	46.19 ± 6.25	36/35	24/25	60	60	JWC + TT	TT	480 mg/d	2 weeks	Clarithromycin + Amoxicillin 2 weeks, Esomeprazole 6–8 weeks	HER, CR, RESR, AR
Deng (2016)	Gastritis	45.8 ± 6.5	45.7 ± 6.4	39/38	36/36	75	74	JWC + TT	TT	320 mg/d	3 weeks	Amoxicillin + Ornidazole + Pantoprazole 3 weeks	CR
Lin (2016)	Gastritis or Peptic ulcer	30.9 ± 5.4	31.2 ± 6.2	28/30	12/10	40	40	JWC + TT	TT	480 mg/d	4 weeks	Esomeprazole + Amoxicillin + Clarithromycin, 4 weeks	HER, CR, RESR
Xu et al. (2016)	Gastritis	41 ± 10.5	43 ± 7.8	38/31	22/29	60	60	JWC + TT	TT	480 mg/d	15 days	Clarithromycin + Tinidazole 1 week, Omeprazole 30 days	HER, CR
Su and Wu (2017)	Peptic ulcer	48.73 ± 12.11	46.28 ± 10.66	36/33	28/30	64	63	JWC + TT	TT	480 mg/d	4 weeks	Omeprazole + Amoxicillin + Clarithromycin 4 weeks	HER
Tao et al. (2017)	Gastritis	43.87 ± 3.8	43.1 ± 3.29	23/25	17/15	40	40	JWC + TT	TT	480 mg/d	4 weeks	Amoxicillin + Clarithromycin + Lansoprazole 4 weeks	HER
Chen (2018)	Peptic ulcer	38.13 ± 12.12	39.56 ± 13.62	15/16	16/20	31	36	JWC + QT	QT	480 mg/d	2 weeks	Clarithromycin + Tinidazole + Bismuth potassium citrate + Rabeprazole 2 weeks	HER, CR
Chen (2018)	Gastritis	48.5 ± 14.08	48.32 ± 11.71	15/14	17/17	32	31	JWC + QT	QT	480 mg/d	14 days	Clarithromycin + Tinidazole + Bismuth potassium citrate + Rabeprazole 2 weeks	HER
Wang (2018a)	Peptic ulcer	56.12 ± 11.24	55.71 ± 12.04	24/26	29/27	53	53	JWC + TT	TT	480 mg/d	3 weeks	Amoxicillin + Clarithromycin 1 week, Lansoprazole 2 weeks	HER
Wang (2018b)	Gastritis	67.52 ± 4.09	68.14 ± 3.29	32/38	28/22	60	60	JWC + TT	TT	480 mg/d	10 days	Pantoprazole + Amoxicillin + Furazolidone 10 days	HER, CR, AR
Zhang et al. (2018)	Gastritis	34.5	38.6	22/26	38/34	60	60	JWC + QT	QT	480 mg/d	2 weeks	Rabeprazole + Amoxicillin + Furazolidone + Bismuth potassium citrate 2 weeks	HER, CR
Zhi and Jiao (2018)	Peptic ulcer	49.19 ± 6.37	48.83 ± 7.13	23/24	20/19	43	43	JWC + TT	TT	480 mg/d	2 weeks	Amoxicillin + Levofloxacin 1 week, Rabeprazole 2 weeks	HER, RESR
Dai and Cheng (2019)	Gastritis	39.94 ± 6.5	35.94 ± 8.5	39/37	29/31	68	68	JWC + TT	TT + Placebo	480 mg/d	4 weeks	Clarithromycin + Pantoprazole + Amoxicillin 4 weeks	HER
Liu (2019)	Gastritis	56.5 ± 15.9	56.3 ± 15.7	36/35	24/25	60	60	JWC + TT	TT	Not reported	3 weeks	Lansoprazole + Amoxicillin + Clarithromycin 3 weeks	HER, HRR, CR, AR
Niu (2019)	Peptic ulcer	40.57 ± 5.31	40.33 ± 5.16	28/28	25/24	53	52	JWC + TT	TT	480 mg/d	4 weeks	Clarithromycin + Amoxicillin + Esomeprazole 4 weeks	HER, CR, RESR
Xu and Yu (2019)	Peptic ulcer	38.14 ± 6.03	37.64 ± 5.81	25/26	21/20	46	46	JWC + QT	QT	480 mg/d	4 weeks	Levofloxacin + Rabeprazole + Amoxicillin + Bismuth potassium citrate 4 weeks	HER, CR, RESR, AR
Zhu (2019)	Peptic ulcer	49.55 ± 5.32	50.12 ± 4.69	24/26	20/18	44	44	JWC + QT	QT	480 mg/d	10 days	Amoxicillin + Pantoprazole + Clarithromycin + Bismuth potassium citrate 10 days	HER
Cheng et al. (2020)	Peptic ulcer	42.58 ± 7.96	41.32 ± 8.48	43/46	37/34	80	80	JWC + TT	TT	480 mg/d	8 weeks	Clarithromycin + Amoxicillin 2 weeks, Esomeprazole 8 weeks	HER, CR, RESR, AR
Hang (2020)	Gastritis	41.1 ± 11.7	41.3 ± 11.67	13/14	17/16	30	30	JWC + QT	QT	480 mg/d	2 weeks	Pantoprazole + Clarithromycin + Amoxicillin + Colloidal bismuth pectin 2 weeks	HER, CR
Yao et al. (2020)	Gastritis or Peptic ulcer	50.7 ± 10.9	51.3 ± 9.7	36/37	60/54	96	91	JWC + QT	QT	480 mg/d	2 weeks	Esmeprazole + Amoxicillin ( Furazolidone) + Clarithromycin + Bismuth Potassium Citrate 2 weeks	HER, AR
Zheng (2020)	Gastritis	41.00 ± 11.66	40.29 ± 22.38	14/16	19/18	33	34	JWC + QT	QT	480 mg/d	2 weeks	Omeprazole Sodium + Bismuth potassium citrate + Amoxicillin + Clarithromycin 2 weeks	HER, AR
Cen (2021)	Peptic ulcer	46.33 ± 6.50	45.68 ± 6.42	24/26	22/20	46	46	JWC + TT	TT	480 mg/d	2 weeks	Amoxicillin + Clarithromycin 2 weeks, Omeprazole 6–8 weeks	HER, CR, RESR
Wang et al. (2021)	Gastritis	42.34 ± 10.67	43.17 ± 12.33	15/16	20/19	35	35	JWC + QT	QT	480 mg/d	2 weeks	Omeprazole + Bismuth potassium citrate + Amoxicillin + Clarithromycin 2 weeks	HER, CR, AR
Xiong et al. (2021)	Peptic ulcer	44.49 ± 5.18	44.77 ± 5.13	25/23	15/17	40	40	JWC + TT	TT	480 mg/d	4 weeks	Clarithromycin + Amoxicillin 2 weeks, Rabeprazole sodium 4 weeks	HER, CR, RESR, AR
Zhu (2021)	Gastritis	41.63 ± 7.8	40.58 ± 7.65	28/30	24/22	52	52	JWC + QT	QT	480 mg/d	1 month	Amoxicillin + Rabeprazole Sodium + Bismuth potassium citrate + Furazolidone, 1 month	HER, AR

Abbreviations: E, experimental group; C, control group; JWC, Jinghua Weikang capsule; TT, triple therapy; QT, quadruple therapy; HER, *H. pylori* eradication rate; HRR, *H. pylori* recurrence rate; CR, cure rate; RESR, response rate; and AR, adverse reaction.

### 3.3 Assessment of risk of bias

**FIGURE 2:**
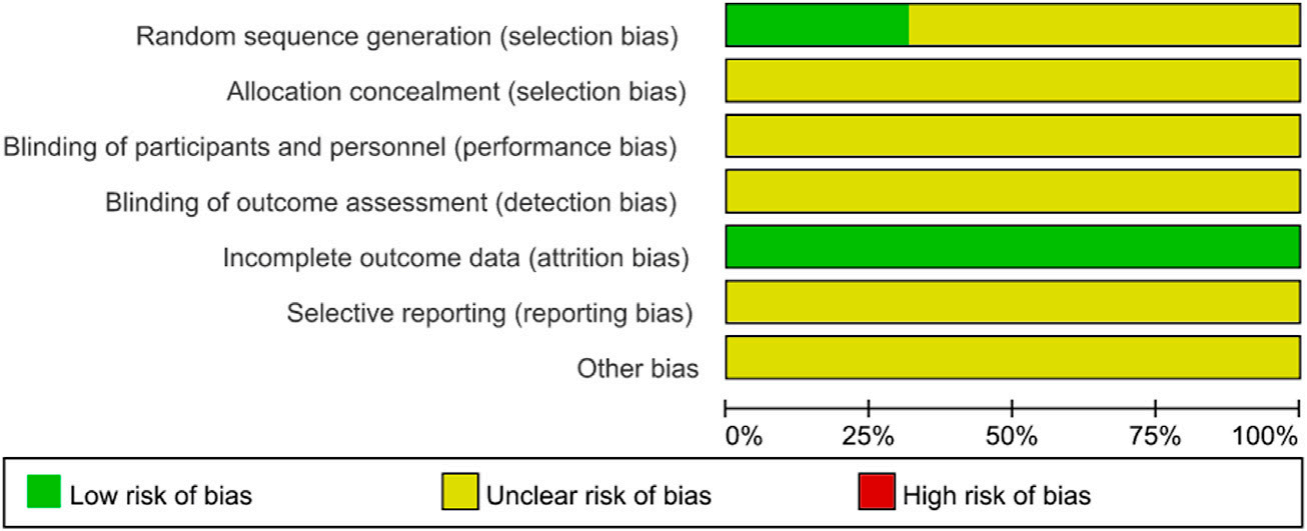
Risk of bias graph.

**FIGURE 3:**
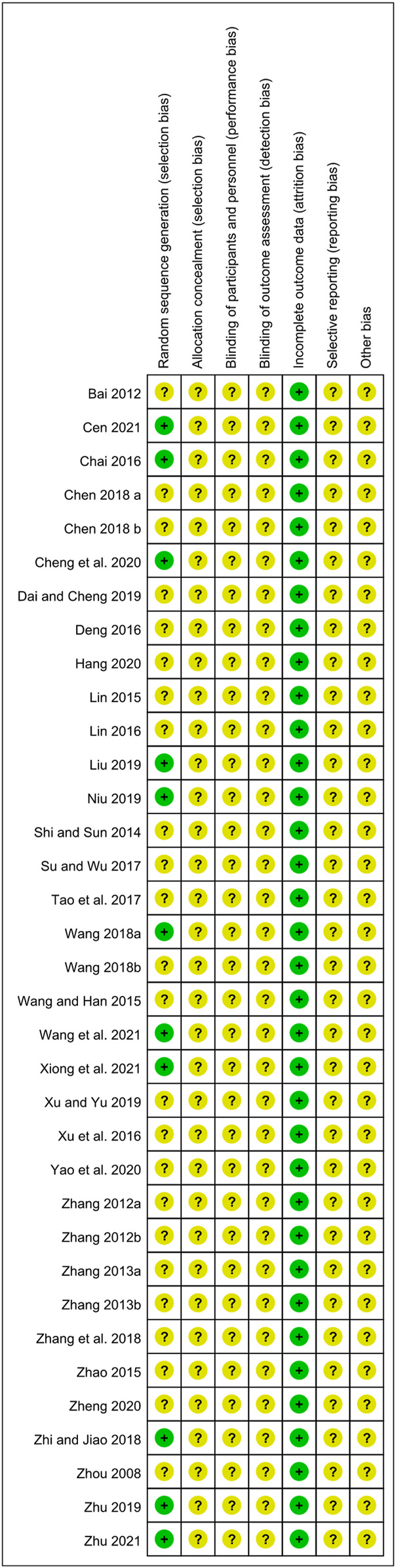
Risk of bias summary.

The results of the risk of bias assessment are presented in Figure 2 and Figure 3. Eleven studies had a low risk of bias for random sequence generation items because they reported specific methods of random sequence generation. Attrition bias was classified as a low level for all included studies because of complete outcome data. The risk of bias for allocation concealment items, performance bias, and detection bias for all included studies was graded as unclear levels due to the lack of sufficient information.

### 3.4 *H. pylori* eradication rate

#### 3.4.1 Treatment duration of 2 weeks

**FIGURE 4:**
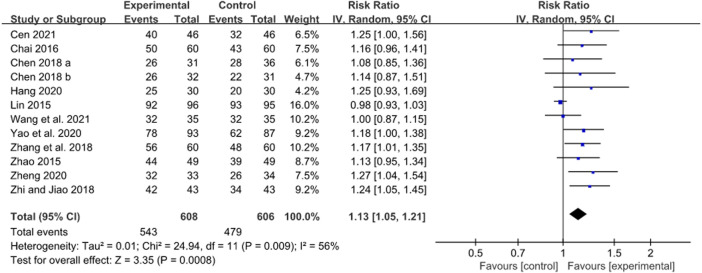
Forest plot of *H. pylori* eradication rate after JWC with the duration of 2 weeks.

**FIGURE 5:**
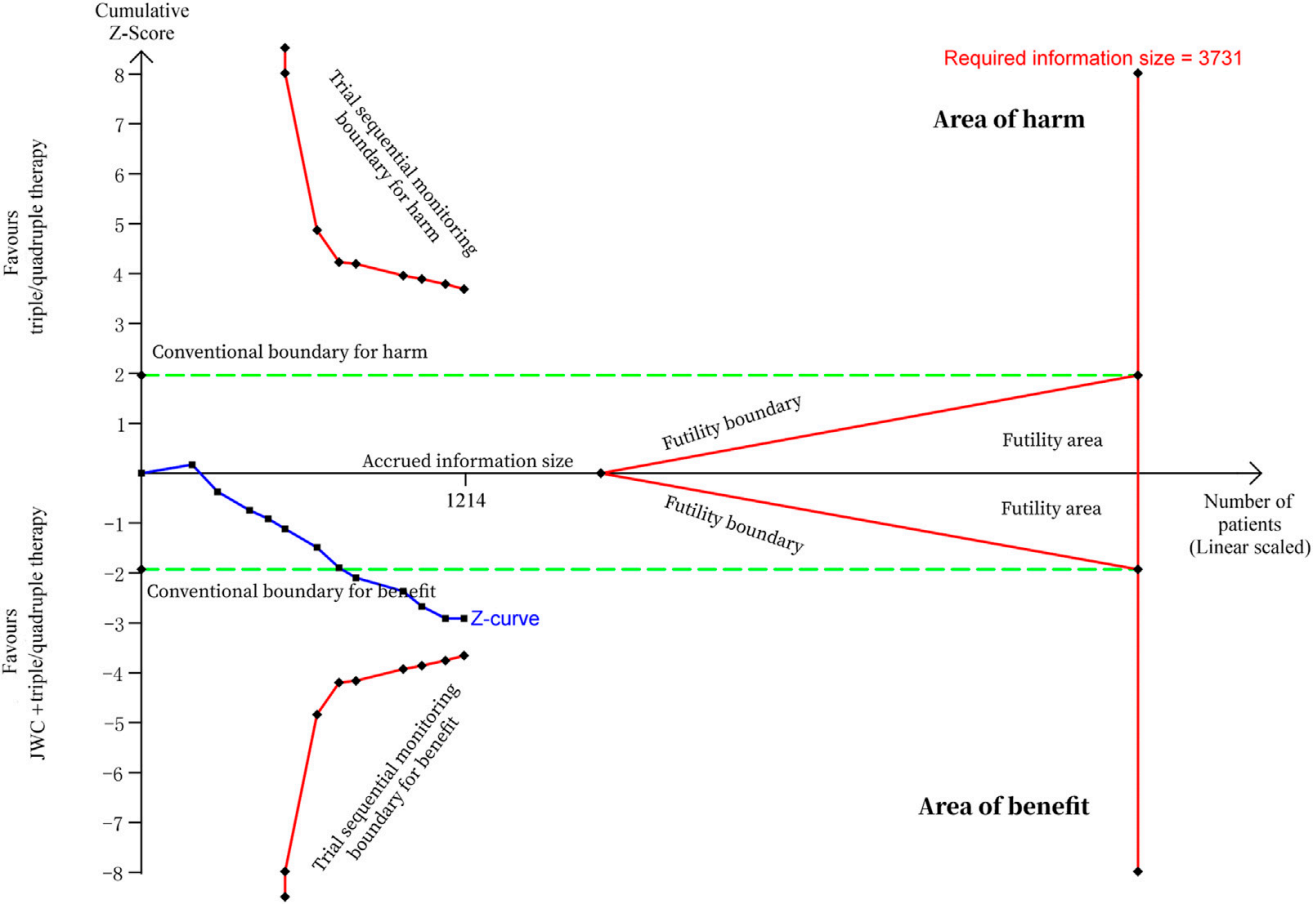
Trial sequential analysis of *H. pylori* eradication rate after JWC with the duration of 2 weeks.

A total of 12 RCTs evaluated the *H. pylori* eradication rate after JWC treatment with the duration of 2 weeks combined with the triple or quadruple therapy (Lin, 2015; Zhao, 2015; Chai, 2016; Chen, 2018; Zhang et al., 2018; Zhi and Jiao, 2018; Hang, 2020; Yao et al., 2020; Zheng, 2020; Cen, 2021; Wang et al., 2021). A pooled result showed that the *H. pylori* eradication rate in JWC combined with the triple or quadruple therapy group was statistically higher than that in the triple or quadruple therapy alone group (N = 12, RR: 1.13, 95% CI: 1.05 to 1.21, *p* = 0.0008, Figure 4). The result from TSA showed that the cumulative Z-curve crossed the conventional boundary for benefit in Figure 5. However, it did not cross the trial sequential monitoring boundary for benefit. This means that firm evidence is not reached and larger-scale trials are still needed. The publication bias might be found because the Z value is equal to 4.26 and *p*-value is less than 0.0001 based on the Harbord test.

Subgroup analyses were conducted based on control interventions (triple and quadruple therapy) and types of diseases (gastritis and peptic ulcer). There was no statistically significant difference in *H. pylori* eradication rate between JWC combined with the triple therapy group and the triple therapy alone group (*N* = 5, RR: 1.13, 95% CI: 1.00 to 1.27, *p* = 0.06). Furthermore, a RCT enrolling patients with gastritis reported a similar result (RR: 0.98, 95% CI: 0.93 to 1.03, *p* = 0.41) (Lin, 2015). However, in patients with peptic ulcer, *H. pylori* eradication rate was statistically higher after JWC combined with the triple therapy compared with the triple therapy alone (*N* = 4, RR: 1.19, 95% CI: 1.09 to 1.30, *p* = 0.0002). A pooled result showed that JWC combined with the quadruple therapy significantly increased the *H. pylori* eradication rate compared with the quadruple therapy alone (*N* = 7, RR: 1.13, 95% CI: 1.06 to 1.22, *p* = 0.0004). A similar result was found in patients with gastritis (*N* = 5, RR: 1.13, 95% CI: 1.03 to 1.24, *p* = 0.008) and not in patients with peptic ulcer (*N* = 1, RR: 1.08, 95% CI: 0.85 to 1.36, *p* = 0.53).

#### 3.4.2 Treatment duration of 3 weeks

Two RCTs reported the *H. pylori* eradication rate, in which patients took JWC for 3 weeks (Wang, 2018a; Liu, 2019). A pooled result showed that *H. pylori* eradication rate was significantly increased after JWC combined with the triple therapy compared with the triple therapy alone (*N* = 2, RR: 1.21, 95% CI: 1.08 to 1.36, *p* = 0.001). Similar findings were also identified in patients with gastritis (*N* = 1, RR: 1.22, 95% CI: 1.04 to 1.42, *p* = 0.01) and peptic ulcer (*N* = 1, RR: 1.20, 95% CI: 1.01 to 1.43, *p* = 0.04).

#### 3.4.3 Treatment duration of 4 weeks

**FIGURE 6:**
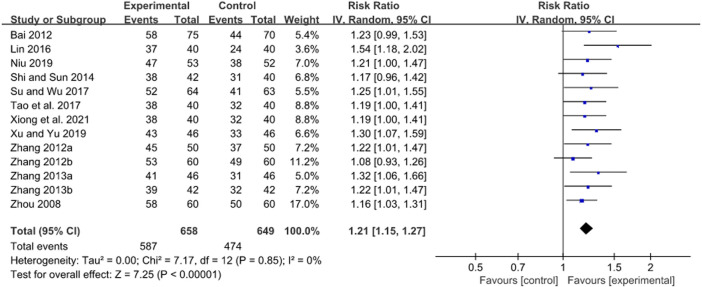
Forest plot of *H. pylori* eradication rate after JWC with the duration of 4 weeks.

**FIGURE 7:**
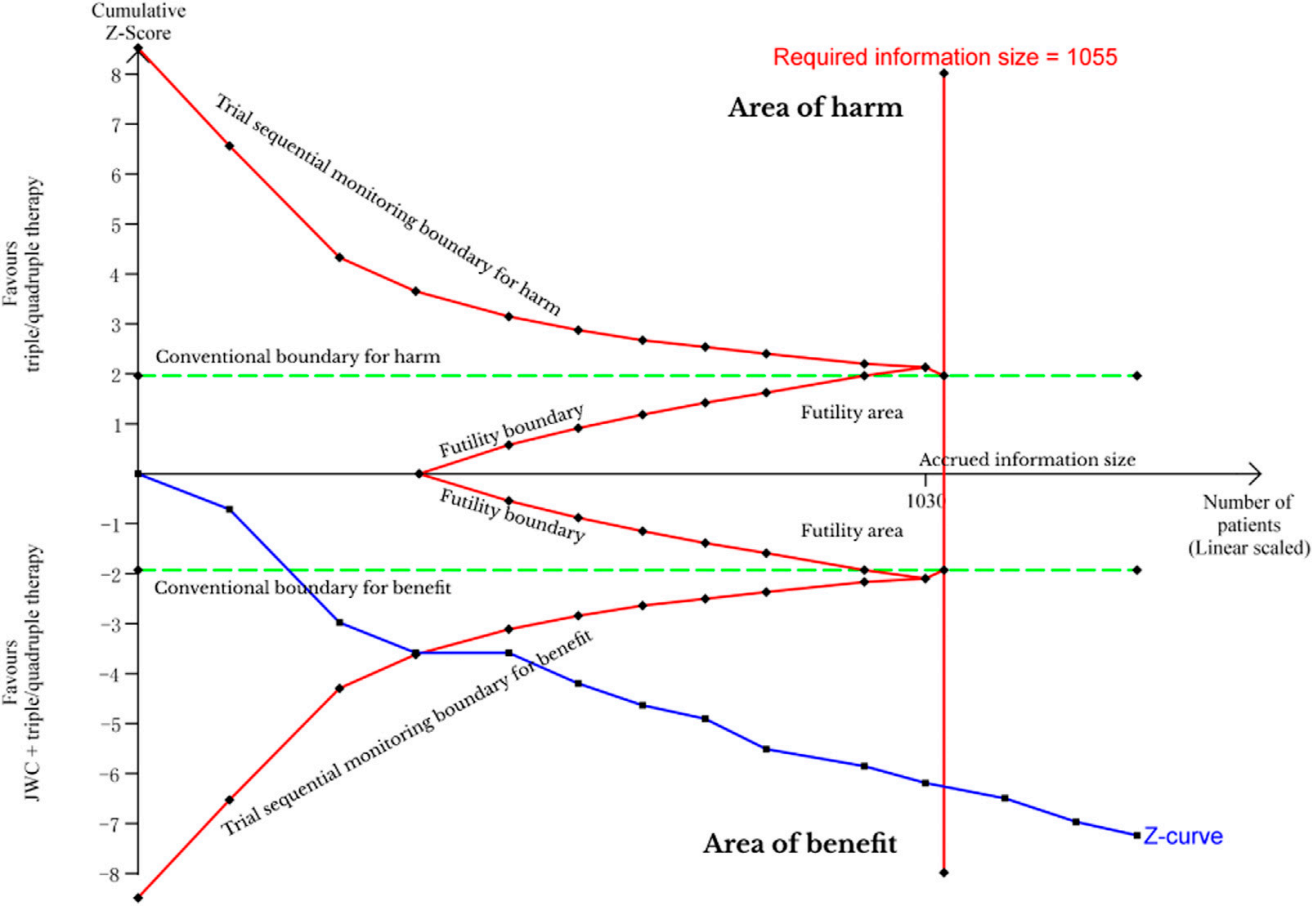
Trial sequential analysis of *H. pylori* eradication rate after JWC with the duration of 4 weeks.

Thirteen RCTs with the JWC treatment for 4 weeks reported the *H. pylori* eradication rate (Zhou, 2008; Zhang, 2012a; Bai, 2012; Zhang, 2012b; Zhang, 2013a; Zhang, 2013b; Shi and Sun, 2014; Lin, 2016; Su and Wu, 2017; Tao et al., 2017; Niu, 2019; Xu and Yu, 2019; Xiong et al., 2021). A pooled result showed that JWC combined with the triple or quadruple therapy could significantly improve the *H. pylori* eradication rate compared with the triple or quadruple therapy alone (*N* = 13, RR: 1.21, 95% CI: 1.15 to 1.27, *p* < 0.00001, Figure 6). The result from TSA showed that the cumulative Z-curve crossed both the conventional boundary for the benefit and the trial sequential monitoring boundary for the benefit in Figure 7. This means that firm evidence is reached and no larger-scale trials are needed. However, the publication bias might be found because the Z value is equal to 2.07 and the *p* value is equal to 0.0382 based on the Harbord test.

Subgroup analyses were conducted based on control interventions (triple and quadruple therapy) and types of diseases (gastritis and peptic ulcer). A pooled result showed a greater increase in *H. pylori* eradication rate after JWC combined with the triple therapy compared with the triple therapy alone (*N* = 12, RR: 1.20, 95% CI: 1.14 to 1.26, *p* < 0.00001). Similar results were also found in patients with gastritis (*N* = 2, RR: 1.20, 95% CI: 1.05 to 1.38, *p* = 0.007) and peptic ulcer (*N* = 9, RR: 1.18, 95% CI: 1.12 to 1.25, *p* < 0.00001). Xu and Yu (2019) reported that JWC combined with the quadruple therapy could significantly increase the *H. pylori* eradication rate compared with the quadruple therapy alone in patients with peptic ulcer (RR: 1.30, 95% CI: 1.07 to 1.59, *p* = 0.008). Dai and Cheng (2019) reported that JWC combined with the triple therapy significantly increased the *H. pylori* eradication rate compared with placebo combined with the triple therapy in patients with gastritis (RR: 1.29, 95% CI: 1.09 to 1.51, *p* = 0.002).

#### 3.4.4 Other treatment durations

Two studies reported that the *H. pylori* eradication rate was significantly increased after JWC combined with the triple therapy compared with the triple therapy alone in patients with gastritis, respectively (*N* = 1, RR: 1.29, 95% CI: 1.06 to 1.57, *p* = 0.010, duration of 10 days; *N* = 1, RR: 1.56, 95% CI: 1.23 to 1.98, *p* = 0.0003, duration of 15 days) (Xu et al., 2016; Wang, 2018b). Two studies showed a greater increase in *H. pylori* eradication rate after JWC combined with the triple therapy compared with the triple therapy alone in patients with peptic ulcer, respectively (*N* = 1, RR: 1.19, 95% CI: 1.09 to 1.29, *p* < 0.0001, duration of 6 weeks; *N* = 1, RR: 1.21, 95% CI: 1.02 to 1.44, *p* = 0.03, duration of 8 weeks) (Wang and Han, 2015; Cheng et al., 2020). Zhu (2019) found that JWC combined with the quadruple therapy could significantly increase the *H. pylori* eradication rate compared with the quadruple therapy alone in patients with peptic ulcer (RR: 1.30, 95% CI: 1.04 to 1.63, *p* = 0.02, duration of 10 days). Zhu (2021) reported that the *H. pylori* eradication rate was significantly increased after JWC combined with the quadruple therapy compared with the quadruple therapy alone in patients with gastritis (RR: 1.23, 95% CI: 1.04 to 1.44, *p* = 0.01, duration of 1 month).

### 3.5 *H. pylori* recurrence rate

Liu (2019) reported a lower *H. pylori* recurrence rate at 6 months after JWC treatment with the duration of 3 weeks combined with the triple therapy compared with the triple therapy alone in patients with gastritis (RR: 0.36, 95% CI: 0.14 to 0.93, *p* = 0.03).

### 3.6 Cure rate

#### 3.6.1 Treatment duration of 2 weeks

**FIGURE 8:**
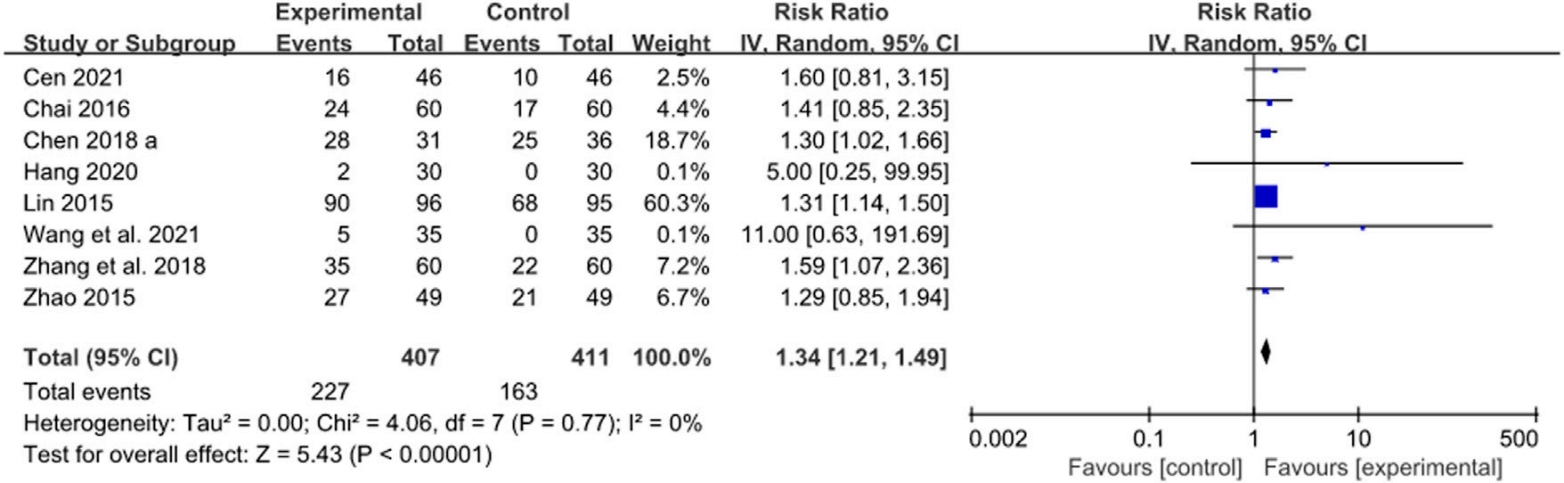
Forest plot of cure rate after JWC with the duration of 2 weeks.

The cure rate of gastritis or peptic ulcer was reported in eight trials, in which patients took JWC for 2 weeks (Lin, 2015; Zhao, 2015; Chai, 2016; Chen, 2018; Zhang et al., 2018; Hang, 2020; Cen, 2021; Wang et al., 2021). A pooled result showed that the cure rate in JWC combined with the triple or quadruple therapy group was statistically higher than that in the triple or quadruple therapy alone group (*N* = 8, RR: 1.34, 95% CI: 1.21 to 1.49, *p* < 0.00001, Figure 8).

Subgroup analyses were conducted based on control interventions (triple and quadruple therapy) and types of diseases (gastritis and peptic ulcer). Compared with the triple therapy alone, JWC combined with the triple therapy could significantly increase the cure rate (*N* = 4, RR: 1.32, 95% CI: 1.17 to 1.50, *p* < 0.00001). Similar results were also found in patients with gastritis (*N* = 1, RR: 1.31, 95% CI: 1.14 to 1.50, *p* = 0.0001) and peptic ulcer (*N* = 3, RR: 1.38, 95% CI: 1.03 to 1.84, *p* = 0.03). Compared with the quadruple therapy alone, JWC combined with the quadruple therapy showed a greater cure rate (*N* = 4, RR: 1.43, 95% CI: 1.10 to 1.86, *p* = 0.007). A similar result was also found in patients with peptic ulcer (*N* = 1, RR: 1.30, 95% CI: 1.02 to 1.66, *p* = 0.04) but not in patients with gastritis (*N* = 3, RR: 1.95, 95% CI: 0.90 to 4.24, *p* = 0.09).

#### 3.6.2 Treatment duration of 3 weeks

Two studies reported a statistically significant increase in the cure rate in JWC combined with the triple therapy group compared with the triple therapy alone group in patients with gastritis (*N* = 2, RR: 1.28, 95% CI: 1.01 to 1.61, *p* = 0.04) (Deng, 2016; Liu, 2019).

#### 3.6.3 Treatment duration of 4 weeks

**FIGURE 9:**
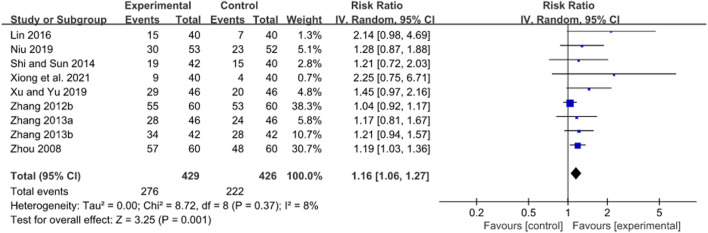
Forest plot of cure rate after JWC with the duration of 4 weeks.

Nine trials compared the cure rate of a combination of JWC with the duration of 4 weeks and the triple/quadruple therapy with the triple/quadruple therapy alone (Zhou, 2008; Zhang, 2012b; Zhang 2013a; Zhang, 2013b; Shi and Sun, 2014; Lin, 2016; Niu, 2019; Xu and Yu, 2019; Xiong et al., 2021). A pooled result showed that the cure rate was statistically higher in the JWC combined with the triple/quadruple therapy group than that in the triple/quadruple therapy alone group (*N* = 9, RR: 1.16, 95% CI: 1.06 to 1.27, *p* = 0.001, Figure 9).

Subgroup analyses were conducted based on control interventions (triple and quadruple therapy) and types of diseases (gastritis and peptic ulcer). Compared with the triple therapy alone, JWC combined with the triple therapy could significantly increase the cure rate (*N* = 8, RR: 1.16, 95% CI: 1.06 to 1.27, *p* = 0.0002). Moreover, JWC combined with the triple therapy could significantly increase the cure rate compared with the triple therapy alone in patients with peptic ulcer (*N* = 7, RR: 1.13, 95% CI: 1.04 to 1.22, *p* = 0.004). Xu and Yu (2019) reported that the cure rate in JWC combined with the quadruple therapy group was higher than that in the quadruple therapy alone group with no statistical significance in patients with peptic ulcer (RR: 1.45, 95% CI: 0.97 to 2.16, *p* = 0.07).

#### 3.6.4 Other treatment durations

Two studies reported that the cure rate in JWC with the duration of 10 days or 15 days combined with the triple therapy group was statistically higher than that in the triple therapy alone group in patients with gastritis, respectively (*N* = 1, RR: 1.25, 95% CI: 1.01 to 1.54, *p* = 0.04, duration of 10 days; *N* = 1, RR: 1.58, 95% CI: 1.20 to 2.08, *p* = 0.0010, duration of 15 days) (Xu et al., 2016; Wang, 2018b). Another study showed that JWC combined with the triple therapy induced a greater cure rate compared with the triple therapy alone in patients with peptic ulcer (RR: 1.62, 95% CI: 1.04 to 2.53, *p* = 0.03, duration of 8 weeks) (Cheng et al., 2020).

### 3.7 Response rate

#### 3.7.1 Treatment duration of 2 weeks

**FIGURE 10:**
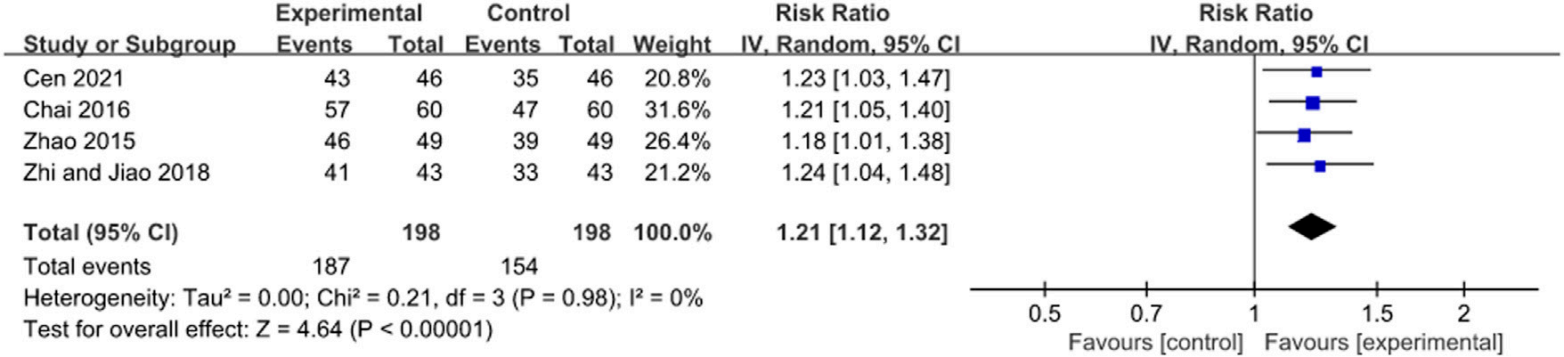
Forest plot of response rate after JWC with the duration of 2 weeks.

A pooled result of 4 RCTs showed that JWC with the duration of 2 weeks combined with the triple therapy could significantly increase the response rate compared with the triple therapy alone in patients with peptic ulcer (RR: 1.21, 95% CI: 1.12 to 1.32, *p* < 0.00001, Figure 10) (Zhao, 2015; Chai, 2016; Zhi and Jiao, 2018; Cen, 2021).

#### 3.7.2 Treatment duration of 4 weeks

**FIGURE 11:**
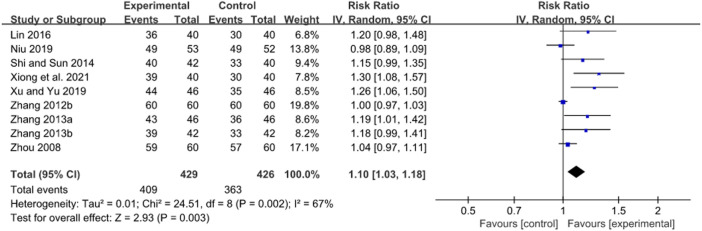
Forest plot of response rate after JWC with the duration of 4 weeks.

A pooled result of nine trials showed that the response rate in JWC combined with the triple or quadruple therapy group was statistically higher than that in the triple or quadruple therapy alone group (RR: 1.10, 95% CI: 1.03 to 1.18, *p* = 0.003, Figure 11) (Zhou, 2008; Zhang, 2012b; Zhang, 2013a; Zhang, 2013b; Shi and Sun, 2014; Lin, 2016; Niu, 2019; Xu and Yu, 2019; Xiong et al., 2021).

A subgroup analysis found that JWC combined with the triple therapy could significantly increase the response rate compared with the triple therapy alone (*N* = 8, RR: 1.08, 95% CI: 1.02 to 1.16, *p* = 0.01). Moreover, JWC combined with the triple therapy could significantly increase the response rate compared with the triple therapy alone in patients with peptic ulcer (*N* = 7, RR: 1.11, 95% CI: 1.02 to 1.21, *p* = 0.02). Xu and Yu (2019) found a greater response rate in JWC combined with the quadruple therapy group compared with the quadruple therapy alone group in patients with peptic ulcer (RR: 1.26, 95% CI: 1.06 to 1.50, *p* = 0.010).

#### 3.7.3 Treatment duration of 8 weeks

Cheng et al. (2020) reported that the response rate in JWC with the duration of 8 weeks combined with the triple therapy group was statistically higher than that in the triple therapy alone group in patients with peptic ulcer (RR: 1.23, 95% CI: 1.07 to 1.42, *p* = 0.004).

### 3.8 Adverse reactions

**FIGURE 12:**
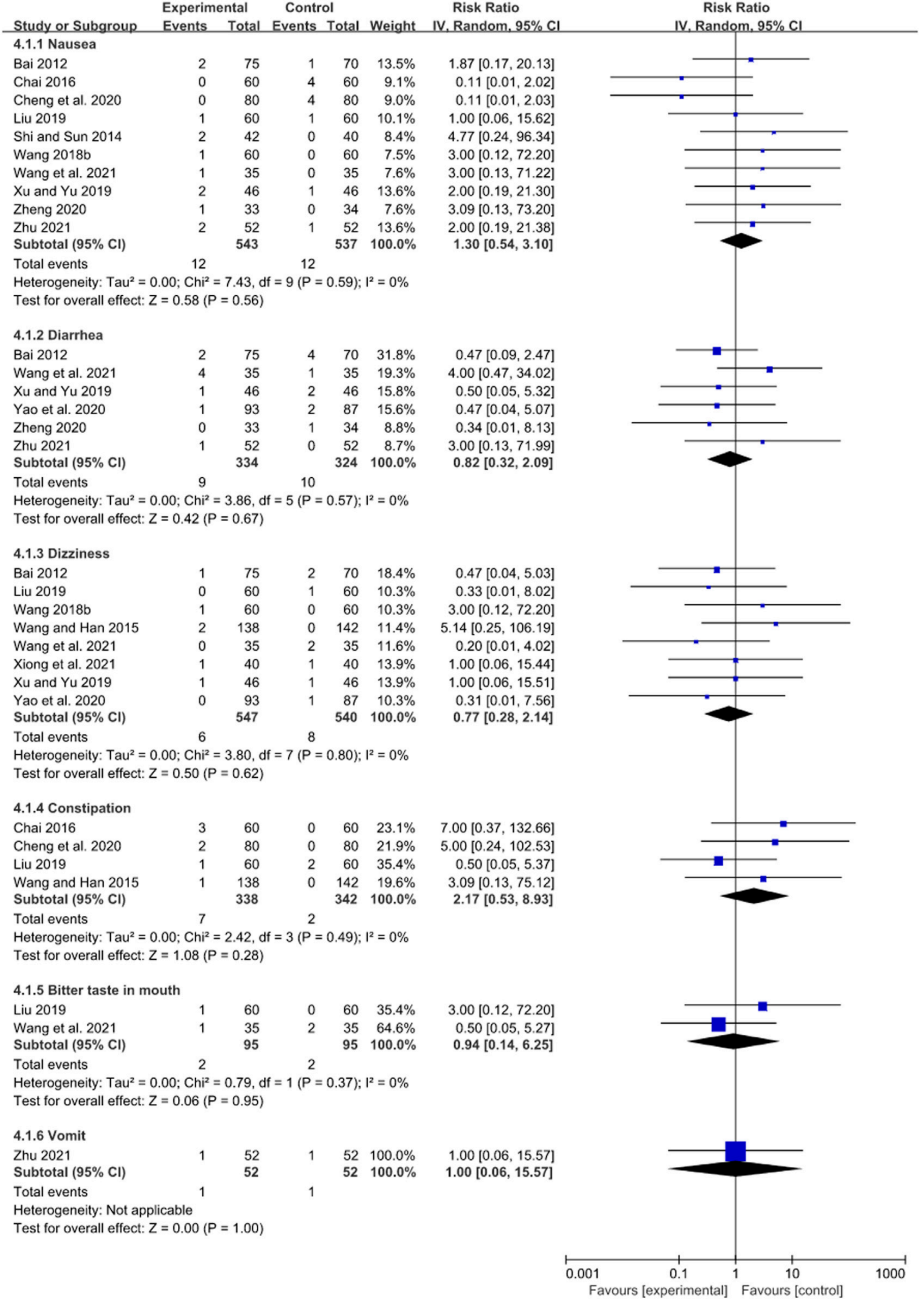
Forest plots of adverse reactions.

Thirteen studies reported adverse reactions (Bai, 2012; Shi and Sun, 2014; Wang and Han, 2015; Chai, 2016; Wang, 2018b; Liu, 2019; Xu and Yu, 2019; Cheng et al., 2020; Yao et al., 2020; Zheng, 2020; Wang et al., 2021; Xiong et al., 2021; Zhu, 2021). The results of the meta-analyses showed no statistically significant differences in the incidence of nausea, diarrhea, dizziness, constipation, vomiting, and bitter taste in the mouth between the two groups in Figure 12.

### 3.9 Quality of evidence

The quality of evidence is presented in Table 3. The quality of evidence on *H. pylori* eradication rate after JWC treatment with the duration of 2 and 4 weeks was graded as very low and low, respectively.

**TABLE 3:** GRADE quality of evidence summary table.

Outcomes	Illustrative comparative risks (95% CI)		RR (95% CI)	No. of participants (studies)	Quality of the evidence
	Assumed risk (Triple/quadruple therapy)	Corresponding risk (JWC + triple/quadruple therapy)			
*H. pylori* eradication rate (JWC for 2 weeks)	790 per 1000	893 per 1000 (830–956)	1.13 (1.05–1.21)	1214 (12 studies)	Very low^a^ ^,^ ^b^ ^,^ ^d^
*H. pylori* eradication rate (JWC for 3 weeks)	761 per 1000	921 per 1000 (822–1000)	1.21 (1.08–1.36)	226 (2 studies)	Very low^a^ ^,^ ^b^ ^,^ ^c^
*H. pylori* eradication rate (JWC for 4 weeks)	730 per 1000	884 per 1000 (840–928)	1.21 (1.15–1.27)	1307 (13 studies)	Low^a^ ^,^ ^b^
*H. pylori* recurrence rate (JWC for 3 weeks)	233 per 1000	84 per 1000 (33–217)	0.36 (0.14–0.93)	120 (1 studies)	Low^a^ ^,^ ^c^
Cure rate (JWC for 2 weeks)	397 per 1000	531 per 1000 (480–591)	1.34 (1.21–1.49)	818 (8 studies)	Low^a^ ^,^ ^b^
Cure rate (JWC for 3 weeks)	403 per 1000	516 per 1000 (407–649)	1.28 (1.01–1.61)	269 (2 studies)	Very low^a^ ^,^ ^b^ ^,^ ^c^
Cure rate (JWC for 4 weeks)	521 per 1000	605 per 1000 (552–662)	1.16 (1.06–1.27)	855 (9 studies)	Low^a^ ^,^ ^b^
Response rate (JWC for 2 weeks)	778 per 1000	941 per 1000 (871–1000)	1.21 (1.12–1.32)	396 (4 studies)	Low^a^ ^,^ ^b^
Response rate (JWC for 4 weeks)	852 per 1000	937 per 1000 (878–1000)	1.10 (1.03–1.18)	855 (9 studies)	Very low^a^ ^,^ ^b^ ^,^ ^d^

Abbreviations: JWC, Jinghua Weikang capsule; CI, confidence interval; and RR, risk ratio.

a Unclear risk of bias due to limitations of blinding and allocation concealment.

b Triple/quadruple therapy, duration, or gastrointestinal disease was inconsistent across studies.

c Only one or two studies were included.

d The confidence interval was wide or I^2^ was more than 50%.

## 4 Discussion

This systematic review critically assessed the efficacy and safety of JWC for *H. pylori* eradication. The main findings are summarized as follows. A pooled result showed that JWC with the duration of 2 weeks combined with the triple/quadruple therapy could significantly increase the *H. pylori* eradication rate compared with the triple/quadruple therapy alone. However, the evidence of benefit was not confirmed by TSA. Subgroup analyses found no statistically significant differences in *H. pylori* eradication rate between JWC combined with the triple therapy and the triple therapy alone in patients with gastritis and between JWC combined with the quadruple therapy and the quadruple therapy alone in patients with peptic ulcer. Another pooled result showed that JWC with the duration of 4 weeks combined with the triple/quadruple therapy could significantly increase the *H. pylori* eradication rate compared with the triple/quadruple therapy alone. The evidence of benefit was confirmed by TSA. Moreover, subgroup analyses also found statistically significant differences in *H. pylori* eradication rate between JWC combined with the triple therapy and the triple therapy alone in patients with gastritis or peptic ulcer, and between JWC combined with the quadruple therapy and the quadruple therapy alone in patients with peptic ulcer. Moreover, one study reported a statistically lower *H. pylori* recurrence rate at 6 months after JWC combined with the triple therapy compared with the triple therapy alone in patients with gastritis. Other results showed that JWC combined with the triple/quadruple therapy could significantly increase the cure rate of gastritis or peptic ulcer and promote peptic ulcer healing compared with the triple/quadruple therapy alone. There were no statistically significant differences in the incidence of adverse reactions between the two groups.

A combination of PPI and antibiotics is typically used for *H. pylori* eradication. For example, clarithromycin triple therapy and bismuth quadruple therapy are recommended according to clinical guidelines (Chey et al., 2017). However, a recent study reported an increased *H. pylori* resistance to antibiotics in most World Health Organization regions (Savoldi et al., 2018). The prevalence of *H. pylori* resistance was also high among children according to a study from Iran (Yousefi-Avarvand et al., 2018). A review reported the reduction of *H. pylori* eradication rate after clarithromycin triple therapy and bismuth quadruple therapy associated with antibiotic resistance (Kim et al., 2015). It poses a great challenge to the selection of treatments for *H. pylori* eradication. A common effort is to seek alternative antibiotics with no *H. pylori* resistance. However, *H. pylori* resistance to many conventional antibiotics has been reported (Kuo et al., 2017; Savoldi et al., 2018). There has been an increased interest in the development of new antibiotics in recent years (Tacconelli et al., 2018). However, few antibiotics have been developed successfully (Tacconelli et al., 2018). Antimicrobial susceptibility testing for *H. pylori* is rarely performed partly due to the lack of standardized testing methods and consensus on antibiotic resistance breakpoints (Li et al., 2022). The salvage therapy may be selected empirically after first-line therapy fails (Chey et al., 2017).

In recent years, some novel therapies have brought benefits to patients with *H. pylori*. Some studies reported the potential of nanotechnology for *H. pylori* eradication (de Souza et al., 2021; Khan et al., 2022). Nonetheless, clinical trials on this topic are still needed. Some systematic reviews showed that probiotics could be considered an adjuvant therapy for *H. pylori* eradication (Yu et al., 2019; Zhou et al., 2019). However, a study reported that probiotics were recommended only for patients with poor compliance to treatments (Shiotani et al., 2017). A recent review suggested that TCM herbs and their active ingredients combined with antibiotics could be considered a novel antibacterial treatment (Su et al., 2020). A systematic review showed that TCM-based therapy could be used as rescue therapy for *H. pylori* eradication (Zhong et al., 2022). Berberine belongs to the isoquinoline alkaloid extracted from Chinese herbal medicine. A systematic review showed that berberine combined with the standard triple therapy could significantly increase the *H. pylori* eradication rate (Hu et al., 2019). The main components of JWC are also extracted from Chinese herbal medicine (Shi et al., 2018). The efficacy of JWC with the duration of 4 weeks for eradicating *H. pylori* is confirmed by both conventional meta-analysis and TSA in the present study.

The efficacy of triple/quadruple therapy for *H. pylori* eradication may be dependent on the intragastric potential of hydrogen (pH) (Shiotani et al., 2017). For example, the instability of clarithromycin was reported at low pH (Erah et al., 1997). An experiment showed that JWC could significantly inhibit the secretion of gastric acid in rats (Xie and Huang, 2001). *H. pylori* are tolerant to multiple antibiotics possibly by forming a biofilm (Yonezawa et al., 2019; Hathroubi et al., 2020). An *in vitro* experiment found that volatile oil extracted from *Dysphania ambrosioides* (L.) Mosyakin & Clemants as the main ingredient of JWC could inhibit the formation of *H. pylori* biofilm (Zhang et al., 2020). A study reported that *Adina pilulifera* (Lam.) Franch. ex Drake as the main ingredient of JWC might prevent *H. pylori* from sticking to the stomach wall by competitively inhibiting the blood group antigen-binding adhesion (BabA) (Hong et al., 2021). Some experiments found that JWC could accelerate peptic ulcer healing by stimulating the secretion of nitric oxide and epidermal growth factor and reducing the endothelin level (Cao et al., 2006; Liang, 2007). Another experiment reported that JWC could inhibit *H. pylori*-induced inflammatory responses by regulating the nuclear factor-kappa B signaling pathway (Shi et al., 2018). The mechanisms of JWC for eradicating *H. pylori* and treating gastritis and peptic ulcer may be explained partly by the abovementioned evidence. Overall, the present study provides new insight into the management of *H. pylori* eradication. JWC can be considered a new complementary treatment to conventional regimens for *H. pylori* eradication.

This systematic review has some minor limitations. Due to the lack of relevant data, the long-term effect of JWC for *H. pylori* eradication is poorly investigated, and the efficacy of JWC versus some novel therapies for *H. pylori* eradication is not compared directly. The unclear risk of bias was identified in the blinding and allocation concealment item. The same triple/quadruple therapies are used between two groups in each included study. However, the specific drugs and durations of triple/quadruple therapies may be different across included studies. The heterogeneity of the meta-analyses may be partly explained by the abovementioned factors.

## 5 Conclusion

The present study suggests that JWC with the duration of 4 weeks can significantly improve *H. pylori* eradication rate and should be considered as a complementary treatment to conventional regimens for *H. pylori* eradication. However, more high-quality RCTs are still needed to confirm these findings.

## Data Availability

The original contributions presented in the study are included in the article/Supplementary Material; further inquiries can be directed to the corresponding authors.

## References

[B1] Bai M. (2012). Effects of Jinghua weikang capsules combined with the standard triple therapy in the treatment of chronic gastritis. J. Drug Eval. 9 (20), 23–25. 10.3969/j.issn.1672-2809.2012.20.005

[B2] Cao M. B. Chang X. M. Dong L. Ren L. (2006). Effect of Jinghua weikang Capsule on concrescence of gastric mucosa in rats with gastric ulcer. Chin. J. New Drugs 1 (16), 1357–1359. 10.3321/j.issn:1003-3734.2006.16.011

[B3] Cen Y. H. (2021). Proton pump inhibitor triple therapy combined with Jinghua Weikang Capsule in the treatment of *Helicobacter pylori*-positive patients with chronic peptic ulcer. Int. Med. Health Guid. News 27 (15), 2359–2362. 10.3760/cma.j.issn.1007-1245.2021.15.037

[B4] Chai Y. P. (2016). Observation on the curative effect of Jinghua weikang capsule combined with triple therapy containing proton pump inhibitor in the treatment of peptic ulcer. China Med. Eng. 24 (04), 110–111. 10.19338/j.issn.1672-2019.2016.04.050

[B5] Chen Q. (2005). Study on GC fingerprint of Jinghua weikang capsule. Fuzhou, China: Strait Pharmaceutical Journal 17 (1), 58–60. 10.3969/j.issn.1006-3765.2005.01.034

[B6] Chen X. (2018). Clinical study on the treatment of chronic gastritis and duodenal bulb ulcer of *helicobacter pylori* by Jinghua weikang combined with quadruple therapy. China: Bengbu Medical College. [Master’s thesis].

[B7] Cheng J. Yu K. T. Tan H. (2020). Efficacy and safety of Jinghua weikang capsule combined with proton pump inhibitor and antibiotic in the treatment of peptic ulcer. Chin. J. Mod. Drug Appl. 14 (09), 150–151. 10.14164/j.cnki.cn11-5581/r.2020.09.069

[B8] Chey W. D. Leontiadis G. I. Howden C. W. Moss S. F. (2017). ACG clinical guideline: Treatment of *Helicobacter pylori* infection. Am. J. Gastroenterol. 112 (2), 212–239. 10.1038/ajg.2016.563 28071659

[B9] Dai M. S. Cheng X. H. (2019). Clinical study on Jinghua weikang soft capsules for chronic gastritis caused by *Helicobacter pylori* . J. New Chin. Med. 51 (11), 110–112. 10.13457/j.cnki.jncm.2019.11.032

[B10] de Souza M. P. C. de Camargo B. A. F. Spósito L. Fortunato G. C. Carvalho G. C. Marena G. D. (2021). Highlighting the use of micro and nanoparticles based-drug delivery systems for the treatment of *Helicobacter pylori* infections. Crit. Rev. Microbiol. 47 (4), 435–460. 10.1080/1040841x.2021.1895721 33725462

[B11] Deng H. H. (2016). Therapeutic effect of integrated traditional Chinese and Western Medicine for *Helicobacter pylori*-infected patients with atrophic gastritis. Cardiovasc. Dis. J. Integr. traditional Chin. West. Med. 4 (26), 181. 10.16282/j.cnki.cn11-9336/r.2016.26.140

[B12] Erah P. O. Goddard A. F. Barrett D. A. Shaw P. N. Spiller R. C. (1997). The stability of amoxycillin, clarithromycin and metronidazole in gastric juice: Relevance to the treatment of *Helicobacter pylori* infection. J. Antimicrob. Chemother. 39 (1), 5–12. 10.1093/jac/39.1.5 9044021

[B13] Hang L. (2020). Clinical research on the treatment of chronic non-atrophic gastritis by *Helicobacter pylori* with Jinghua weikang capsule combined with cuadruple combination therapy. China: Nanjing University of Chinese Medicine. [Master’s thesis].

[B14] Hathroubi S. Zerebinski J. Clarke A. Ottemann K. M. (2020). *Helicobacter pylori* biofilm confers antibiotic tolerance in part via A protein-dependent mechanism. Antibiot. (Basel) 9 (6), E355. 10.3390/antibiotics9060355 PMC7345196 32599828

[B15] Higgins J. P. T. Thomas J. Chandler J. Cumpston M. Li T. Page M. J. (2022). Cochrane handbook for systematic reviews of interventions version 6.3 (updated february 2022). Cochrane, 2022. Available from www.training.cochrane.org/handbook (Accessed March 15, 2022).

[B16] Hong S. W. Yang Y. Xu D. L. Cao Q. Q. Yin H. L. Xia Z. Y. (2021). Systematic pharmacological methodology-based investigation on interventional mechanism of Jinghua weikang capsule protecting against *Helicobacter pylori*-induced inflammatory responses. J. Anhui Sci. Technol. Univ. 35 (02), 57–66. 10.19608/j.cnki.1673-8772.2017.0899

[B17] Hooi J. K. Y. Lai W. Y. Ng W. K. Suen M. M. Y. Underwood F. E. Tanyingoh D. (2017). Global prevalence of *Helicobacter pylori* infection: Systematic review and meta-analysis. Gastroenterology 153 (2), 420–429. 10.1053/j.gastro.2017.04.022 28456631

[B18] Hu Q. Peng Z. Li L. Zou X. Xu L. Gong J. (2019). The efficacy of berberine-containing quadruple therapy on Helicobacter pylori eradication in China: A systematic review and meta-analysis of randomized clinical trials. Front. Pharmacol. 10, 1694. 10.3389/fphar.2019.01694 32116685 PMC7010642

[B19] IARC Working Group on the Evaluation of Carcinogenic Risks to Humans (2012). Biological agents. Volume 100 B. A review of human carcinogens. Switzerland: World Health Organization Press.

[B20] Khan S. Sharaf M. Ahmed I. Khan T. U. Shabana S. Arif M. (2022). Potential utility of nano-based treatment approaches to address the risk of *Helicobacter pylori* . Expert Rev. anti. Infect. Ther. 20 (3), 407–424. 10.1080/14787210.2022.1990041 34658307

[B21] Kim S. Y. Choi D. J. Chung J. W. (2015). Antibiotic treatment for *Helicobacter pylori*: Is the end coming? World J. Gastrointest. Pharmacol. Ther. 6 (4), 183–198. 10.4292/wjgpt.v6.i4.183 26558152 PMC4635158

[B22] Kuo Y. T. Liou J. M. El-Omar E. M. Wu J. Y. Leow A. H. R. Goh K. L. (2017). Primary antibiotic resistance in *Helicobacter pylori* in the asia-pacific region: A systematic review and meta-analysis. Lancet. Gastroenterol. Hepatol. 2 (10), 707–715. 10.1016/s2468-1253(17)30219-4 28781119

[B23] Laxminarayan R. Van Boeckel T. Frost I. Kariuki S. Khan E. A. Limmathurotsakul D. (2020). The lancet infectious diseases commission on antimicrobial resistance: 6 years later. Lancet. Infect. Dis. 20 (4), e51–e60. 10.1016/s1473-3099(20)30003-7 32059790

[B24] Lee Y. C. Chiang T. H. Chou C. K. Tu Y. K. Liao W. C. Wu M. S. (2016). Association between *Helicobacter pylori* eradication and gastric cancer incidence: A systematic review and meta-analysis. Gastroenterology 150 (5), 1113–1124. e1115. 10.1053/j.gastro.2016.01.028 26836587

[B25] Li H. Shen Y. Song X. Tang X. Hu R. Marshall B. J. (2022). Need for standardization and harmonization of *Helicobacter pylori* antimicrobial susceptibility testing. Helicobacter 27 (2), e12873. 10.1111/hel.12873 35151236

[B26] Li R. J. Dai Y. Y. Qin C. Huang G. R. Qin Y. C. Huang Y. Y. (2021b). Application of traditional Chinese medicine in treatment of *Helicobacter pylori* infection. World J. Clin. Cases 9 (35), 10781–10791. 10.12998/wjcc.v9.i35.10781 35047590 PMC8678867

[B27] Li Y. Li X. Tan Z. (2021a). An overview of traditional Chinese medicine therapy for *Helicobacter pylori*-related gastritis. Helicobacter 26 (3), e12799. 10.1111/hel.12799 33765344

[B28] Liang W. Y. (2007). Effect of Jinghuaweikang Capsule on the levels of NO, NOS and ET in the gastric mucous of rats with gastric ulcer. China: Beijing University of Chinese Medicine. [Master’s thesis].

[B29] Lin R. F. (2016). Clinical observation on treating 40 *Helicobacter pylori*-infected cases with Jinghua weikang capsule combined with Western medicine. Clin. J. Chin. Med. 8 (01), 81–83. 10.3969/j.issn.1674-7860.2016.01.041

[B30] Lin S. Q. (2015). Clinical study of Jinghuaweikang capsule in the adjuvant treatment of *Helicobacter pylori*-positive patients with chronic gastritis. J. New Chin. Med. 47 (08), 73–74. 10.13457/j.cnki.jncm.2015.08.033

[B31] Liu L. (2019). Effects of Jinghua Weikang capsule combined with triple therapy in the treatment of *Helicobacter pylori*-positive patients with verrucous gastritis. Chin. J. Prim. Med. Pharm. 26 (06), 641–645. 10.3760/cma.j.issn.1008-6706.2019.06.001

[B32] Machlowska J. Baj J. Sitarz M. Maciejewski R. Sitarz R. (2020). Gastric cancer: Epidemiology, risk factors, classification, genomic characteristics and treatment strategies. Int. J. Mol. Sci. 21 (11), E4012. 10.3390/ijms21114012 32512697 PMC7312039

[B33] Malfertheiner P. Megraud F. O'Morain C. A. Gisbert J. P. Kuipers E. J. Axon A. T. (2017). Management of *Helicobacter pylori* infection-the maastricht V/florence consensus report. Gut 66 (1), 6–30. 10.1136/gutjnl-2016-312288 27707777

[B34] Malfertheiner P. Megraud F. O'Morain C. Bazzoli F. El-Omar E. Graham D. (2007). Current concepts in the management of *Helicobacter pylori* infection: The maastricht III consensus report. Gut 56 (6), 772–781. 10.1136/gut.2006.101634 17170018 PMC1954853

[B35] Niu L. P. (2019). Jinghua weikang capsule combined with esomeprazole triple therapy in the treatment of *Helicobacter pylori*-positive patients with gastric ulcer. Henan Med. Res. 28 (12), 2249–2251. CNKI:SUN:HNYX.0.2019-12-066.

[B36] Page M. J. McKenzie J. E. Bossuyt P. M. Boutron I. Hoffmann T. C. Mulrow C. D. (2021). The PRISMA 2020 statement: An updated guideline for reporting systematic reviews. Bmj 372, n71. 10.1136/bmj.n71 33782057 PMC8005924

[B37] Randel A. (2018). *H. pylori* infection: ACG updates treatment Recommendations. Am. Fam. Physician 97 (2), 135–137. 29365220

[B38] Savoldi A. Carrara E. Graham D. Y. Conti M. Tacconelli E. (2018). Prevalence of antibiotic resistance in *Helicobacter pylori*: A systematic review and meta-analysis in world health organization regions. Gastroenterology 155 (5), 1372–1382. e1317. 10.1053/j.gastro.2018.07.007 29990487 PMC6905086

[B39] Shi Y. Y. Sun J. (2014). Clinical observation of Jinghua weikang capsule for peptic ulcerCNKI:SUN:YCGC. China Med. Eng. 22 (12), 114. 10.2014-12-093.

[B40] Shi Z. M. Ye H. Yu J. Zhang X. Cheng H. Li J. (2018). Jinghua Weikang capsule protects against *Helicobacter pylori*-induced inflammatory responses via the nuclear factor-kappa B signaling pathway. J. Traditional Chin. Med. 38 (3), 366–372. 10.1016/j.jtcm.2018.03.001 32185968

[B41] Shiotani A. Lu H. Dore M. P. Graham D. Y. (2017). Treating *Helicobacter pylori* effectively while minimizing misuse of antibiotics. Cleve. Clin. J. Med. 84 (4), 310–318. 10.3949/ccjm.84a.14110 28388387 PMC6905081

[B42] Su T. Qiu Y. Hua X. Ye B. Luo H. Liu D. (2020). Novel opportunity to reverse antibiotic resistance: To explore traditional Chinese medicine with potential activity against antibiotics-resistance bacteria. Front. Microbiol. 11, 610070. 10.3389/fmicb.2020.610070 33414777 PMC7782309

[B43] Su Y. S. Wu F. (2017). Efficacy of Jinghua weikang combined with triple therapy on *Helicobacter pylori*-positive patients with peptic ulcer. China Med. Pharm. 7 (21), 215–217. 10.3969/j.issn.2095-0616.2017.21.064

[B44] Tacconelli E. Carrara E. Savoldi A. Harbarth S. Mendelson M. Monnet D. L. (2018). Discovery, research, and development of new antibiotics: The WHO priority list of antibiotic-resistant bacteria and tuberculosis. Lancet. Infect. Dis. 18 (3), 318–327. 10.1016/s1473-3099(17)30753-3 29276051

[B45] Tao H. Y. Lu Y. Huang X. Y. (2017). Clinical observation of Jinghuaweikang capsule in the treatment of *Helicobacter pylori*-positive patients with chronic gastritis. Shanxi J. Traditional Chin. Med. 38 (02), 159–160. 10.3969/j.issn.1000-7369.2017.02.009

[B46] Thorlund K. Devereaux P. J. Wetterslev J. Guyatt G. Ioannidis J. P. Thabane L. (2009). Can trial sequential monitoring boundaries reduce spurious inferences from meta-analyses? Int. J. Epidemiol. 38 (1), 276–286. 10.1093/ije/dyn179 18824467

[B47] Torre L. A. Bray F. Siegel R. L. Ferlay J. Lortet-Tieulent J. Jemal A. (2015). Global cancer statistics, 2012. Ca. Cancer J. Clin. 65 (2), 87–108. 10.3322/caac.21262 25651787

[B48] Wang H. J. (2018a). Effect of triple therapy combined with Jinghua Weikang Capsule on improvement of duodenal ulcer symptoms and *Helicobacter pylori* eradication rate. Inn. Mong. Med. J. 50 (05), 596–598. 10.16096/J.cnki.nmgyxzz.2018.50.05.040

[B49] Wang J. H. Han Y. C. (2015). Clinical analysis of Jinghua weikang capsule in the treatment of 138 patients with peptic ulcer. J. North Pharm. 12 (08), 168–169. CNKI: SUN:BFYX.0.2015-08-132.

[B50] Wang R. X. Zhang S. S. Zhou Q. (2021). The research of curative effect of patients with chronic gastritis associated with *Helicobacter pylori* treated by Jinghua Weikang Capsule combined with standard quadruple therapy. Chin. J. Integr. Traditional West. Med. Dig. 29 (09), 610–614. 10.3969/j.issn.1671-038X.2021.09.03

[B51] Wang Y. G. (2018b). To observe the clinical efficacy of Jinghua weikang capsule combined with triple therapy in the treatment of *Helicobacter pylori*-infected chronic gastritis. Qinghai Med. J. 48 (12), 59–60. CNKI:SUN:QHYZ.0.2018-12-026.

[B52] Wetterslev J. Jakobsen J. C. Gluud C. (2017). Trial Sequential Analysis in systematic reviews with meta-analysis. BMC Med. Res. Methodol. 17 (1), 39. 10.1186/s12874-017-0315-7 28264661 PMC5397700

[B53] Wetterslev J. Thorlund K. Brok J. Gluud C. (2008). Trial sequential analysis may establish when firm evidence is reached in cumulative meta-analysis. J. Clin. Epidemiol. 61 (1), 64–75. 10.1016/j.jclinepi.2007.03.013 18083463

[B54] Xie Z. J. Huang M. X. (2001). Inhibitory effect of Jinghua weikang Capsule on experimental gastric ulcer and *Helicobacter pylori* . Chin. J. New Drugs 1 (03), 221–223. 10.3321/j.issn:1003-3734.2001.03.024

[B55] Xiong Y. F. Yuan S. H. Zhang Q. H. (2021). Clinical observation of Jinghua Weikang capsules combined with Rabeprazole triple therapy in the treatment of *Helicobacter pylori*-related gastric ulcer. Med. Forum 25 (35), 5050–5052. 10.19435/j.1672-1721.2021.35.006

[B56] Xu L. P. Yu H. S. (2019). Efficacy of Jinghua weikang capsule in the treatment of *Helicobacter pylori*-positive patients with gastric ulcer. J. Shanxi Med. Coll. Continuing Educ. 29 (04), 74–76.

[B57] Xu Y. M. Lin J. H. Liang X. H. Xie Y. H. Cheng W. H. (2016). Observation on the efficacy of Jinghua weikang capsule in the treatment of chronic gastritis and eradication of *Helicobacter pylori* . J. Intern. Intensive Med. 22 (01), 41–42. 10.11768/nkjwzzzz20160115

[B58] Yao G. P. Li Q. X. Duan X. H. Yin H. K. (2020). A randomized controlled study of Jinghua weikang capsule combined with quadruple therapy in the treatment of *Helicobacter pylori* infection. Mod. Dig. Intervention 25 (07), 922–925. 10.3969/j.issn.1672-2159.2020.07.020

[B59] Yonezawa H. Osaki T. Hojo F. Kamiya S. (2019). Effect of *Helicobacter pylori* biofilm formation on susceptibility to amoxicillin, metronidazole and clarithromycin. Microb. Pathog. 132, 100–108. 10.1016/j.micpath.2019.04.030 31034965

[B60] Yousefi-Avarvand A. Vaez H. Tafaghodi M. Sahebkar A. H. Arzanlou M. Khademi F. (2018). Antibiotic resistance of *Helicobacter pylori* in Iranian children: A systematic review and meta-analysis. Microb. Drug Resist. 24 (7), 980–986. 10.1089/mdr.2017.0292 29227738

[B61] Yu M. Zhang R. Ni P. Chen S. Duan G. (2019). Efficacy of lactobacillus-supplemented triple therapy for *H. pylori* eradication: A meta-analysis of randomized controlled trials. PLoS One 14 (10), e0223309. 10.1371/journal.pone.0223309 31577828 PMC6774518

[B62] Zhang E. E. Ye H. Jia X. F. Huang Q. Y. Zhang X. Z. Liu Y. (2020). Effect of *Chenopodium ambrosioides* L. On biofilm formation of *Helicobacter pylori in vitro* . Chin. J. Integr. Traditional West. Med. 40 (10), 1241–1245. 10.7661/j.cjim.20200904.334

[B63] Zhang H. Y. (2013a). Jinghua weikang capsule combined with triple therapy in the treatment of 46 *Helicobacter pylori*-positive patients with peptic ulcer. China Pharm. 22 (16), 109. 10.3969/j.issn.1006-4931.2013.16.062

[B64] Zhang H. Y. Liu J. Liu W. P. Liu Y. Lin H. Y. Li X. L. (2018). Clinical observation of 60 patients with Verrucous Gastritis Treated with traditional Chinese medicine via *Helicobacter pylori* eradication. Mil. Med. J. Southeast China 20 (01), 70–72. 10.3969/j.issn.1672-271X.2018.01.016

[B65] Zhang L. L. (2013b). Observation on the curative effect of Jinghua weikang capsule plus quadruple therapy in the treatment of peptic ulcer. Med. J. Chin. People's Health 25 (09), 11–12. 10.3969/j.issn.1672-0369.2013.09.001

[B66] Zhang M. M. Qian W. Qin Y. Y. He J. Zhou Y. H. (2015). Probiotics in *Helicobacter pylori* eradication therapy: A systematic review and meta-analysis. World J. Gastroenterol. 21 (14), 4345–4357. 10.3748/wjg.v21.i14.4345 25892886 PMC4394097

[B67] Zhang W. M. (2012a). Jinghua Weikang Capsule Combined with triple therapy to eradicate *Helicobacter pylori* in patients with gastroduodenal ulcer. Chin. J. Mod. Drug Appl. 6 (03), 65–67. 10.14164/j.cnki.cn11-5581/r.2012.03.070

[B68] Zhang Y. Zhang J. Huang X. Zhou X. Wu H. Guo S. (2012b). Observation on the curative effect of Jinghua weikang capsule combined with triple therapy for *Helicobacter pylori*-positive patients with duodenal ulcer. Small 9 (16), 154–159. 10.1002/smll.201101695

[B69] Zhao D. Fan Y. Gao F. Yang T. m. (2015). Curative effect of Triple therapy combined Jinghua Weikang capsule for *Helicobacter pylori* eradication in patients with gastric ulcer. Anal. Chim. Acta 34 (01), 131–137. 10.1016/j.aca.2015.06.053

[B70] Zheng L. (2020). Clinical observation of Jinghua weikang capsule combined with bismuth agent in the treatment of *Helicobacter prlori*-associated aastritis. China: Hubei University of Chinese Medicine. [Master’s thesis].

[B71] Zhi H. Jiao K. F. (2018). Effect of Jinghua Weikang Capsule Combined with rabeprazole triple therapy in the treatment of *Helicobacter pylori* positive-patients with duodenal ulcer. Henan Med. Res. 27 (19), 3523–3524. 10.3969/j.issn.1004-437X.2018.19.030

[B72] Zhong M. F. Li J. Liu X. L. Gong P. Zhang X. T. (2022). TCM-based therapy as a rescue therapy for Re-eradication of *Helicobacter pylori* infection: A systematic review and meta-analysis. Evid. Based. Complement. Altern. Med. 2022, 5626235. 10.1155/2022/5626235 PMC8894008 35251209

[B73] Zhou B. G. Chen L. X. Li B. Wan L. Y. Ai Y. W. (2019). *Saccharomyces boulardii* as an adjuvant therapy for *Helicobacter pylori* eradication: A systematic review and meta-analysis with trial sequential analysis. Helicobacter 24 (5), e12651. 10.1111/hel.12651 31414551

[B74] Zhou Y. (2008). Observation on the curative effect of Jinghua weikang capsule combined with triple therapy for peptic ulcer. Mod. J. Integr. Traditional Chin. West. Med. 17 (28), 4426–4427. 10.3969/j.issn.1008-8849.2008.28.041

[B75] Zhu G. L. (2019). Efficacy of Jinghua weikang capsule combined with pantoprazole quadruple therapy for 44 *Helicobacter pylori*-positive patients with duodenal ulcer. J. North Pharm. 16 (01), 106–107. CNKI:SUN:BFYX.0.2019-01-083.

[B76] Zhu Y. C. (2021). Efficacy of Jinghua Weikang capsule combined with rabeprazole quadruple therapy in the treatment of *Helicobacter pylori*-infected patients with Verrucous Gastritis. Pract. Clin. J. Integr. Traditional Chin. West. Med. 21 (06), 73–74. 10.13638/j.issn.1671-4040.2021.06.036

[B77] Zhu Y. H. Li W. Han J. P. Chu Y. Wang X. Y. (2012). Simultaneous determination of ascaridole, p-cymene and α-terpinene in Jinghua weikang capsule by GC. Chin. J. New Drugs 1 (07), 737–739. CNKI:SUN:ZXYZ.0.2012-07-011.

