# Supplementary material for: Jinghua Weikang capsule for helicobacter pylori eradication: A systematic review and meta-analysis with trial sequential analysis
Source: Front Pharmacol. 2022 Sep 26;13:959184. doi: 10.3389/fphar.2022.959184 (PMC9549166; doi:10.3389/fphar.2022.959184)
Supplement: Supplementary file 1 [file Table1.DOCX]

**PubMed:**

#1 ("Helicobacter pylori"[Title/Abstract] OR "H. pylori"[Title/Abstract] OR "Helicobacter infection"[Title/Abstract] OR helicobacter[Title/Abstract] OR pylori[Title/Abstract] OR pyloridis[Title/Abstract] OR "HP"[Title/Abstract] OR campylobacter[Title/Abstract])

#2 ("Jinghuaweikang"[Title/Abstract] OR "Jinghua weikang"[Title/Abstract] OR "Jing Hua Wei Kang"[Title/Abstract])

#3 ("randomized controlled trial"[Title/Abstract] OR "controlled clinical trial"[Title/Abstract] OR randomized[Title/Abstract] OR placebo[Title/Abstract] OR randomly[Title/Abstract] OR trial[Title/Abstract])

#4 #1 AND #2 AND #3

**Embase:**

#1 Helicobacter/exp OR 'Helicobacter infection'/exp

#2 helicobacter*:ab,ti,kw OR pylori:ab,ti,kw OR pyloridis:ab,ti,kw OR HP:ab,ti,kw OR campylobacter:ab,ti,kw

#3 #1 OR #2

#4 Jinghuaweikang:ab,ti,kw OR 'Jinghua weikang':ab,ti,kw OR 'Jing Hua Wei Kang':ab,ti,kw

#5 'randomized controlled trial':ab,ti,kw OR 'controlled clinical trial':ab,ti,kw OR randomized:ab,ti,kw OR placebo:ab,ti,kw OR randomly:ab,ti,kw OR trial:ab,ti,kw

#6 #3 AND #4 AND #5

**Web of Science:**

#1 TS=(Helicobacter pylori) OR TS=(H. pylori) OR TS=(Helicobacter infection) OR TS=(helicobacter) OR TS=(pylori) OR TS=(pyloridis) OR TS=(campylobacter)

#2 TS=(Jinghuaweikang) OR TS=(Jinghua weikang) OR TS=(Jing Hua Wei Kang)

#3 TS=(randomized controlled trial) OR TS=(controlled clinical trial) OR TS=(randomized) OR TS=(placebo) OR TS=(randomly) OR TS=(trial)

#4 #1 AND #2 AND #3

**Cochrane library:**

#1 helicobacter*:ti,ab,kw OR 'Helicobacter infection':ti,ab,kw OR pylori:ti,ab,kw OR pyloridis:ti,ab,kw OR HP:ti,ab,kw OR campylobacter:ti,ab,kw

#2 Jinghuaweikang:ti,ab,kw OR 'Jinghua weikang':ti,ab,kw OR 'Jing Hua Wei Kang':ti,ab,kw

#3 'randomized controlled trial':ti,ab,kw OR 'controlled clinical trial':ti,ab,kw OR randomized:ti,ab,kw OR placebo:ti,ab,kw OR randomly:ti,ab,kw OR trial:ti,ab,kw

#4 #1 AND #2 AND #3

**China National Knowledge Infrastructure (CNKI)**

(SU=幽门螺杆菌 OR SU=幽门螺旋杆菌 OR SU=幽门弯曲菌 OR SU=Hp感染 OR SU=Hp阳性) AND (SU=荆花胃康) AND (SU=试验 OR SU=观察 OR SU=随机 OR SU=对照)

**Wanfang Digital Periodicals (WANFANG)**

(主题词:幽门螺杆菌 OR 主题词:幽门螺旋杆菌 OR 主题词:幽门弯曲菌 OR 主题词:Hp感染 OR 主题词:Hp阳性) AND (主题词:荆花胃康) AND (主题词:试验 OR 主题词:观察 OR 主题词:随机 OR 主题词:对照)

**Chinese Science and Technology Periodicals (VIP) database**

U=(幽门螺杆菌 OR 幽门螺旋杆菌 OR 幽门弯曲菌 OR Hp感染 OR Hp阳性) AND U=(荆花胃康) AND U=(试验 OR 观察 OR 随机 OR 对照)
